# Chemokines Associated with Pathologic Responses to Orthopedic Implant Debris

**DOI:** 10.3389/fendo.2017.00005

**Published:** 2017-01-19

**Authors:** Nadim J. Hallab, Joshua J. Jacobs

**Affiliations:** ^1^Department of Orthopedics, Rush University Medical Center, Chicago, IL, USA

**Keywords:** implant debris, inflammasome, orthopedics, allergy, chemokines, CXC

## Abstract

Despite the success in returning people to health saving mobility and high quality of life, the over 1 million total joint replacements implanted in the US each year are expected to eventually fail after approximately 15–25 years of use, due to slow progressive subtle inflammation to implant debris compromising the bone implant interface. This local inflammatory pseudo disease state is primarily caused by implant debris interaction with innate immune cells, i.e., macrophages. This implant debris can also activate an adaptive immune reaction giving rise to the concept of implant-related metal sensitivity. However, a consensus of studies agree the dominant form of this response is due to innate reactivity by macrophages to implant debris danger signaling (danger-associated molecular pattern) eliciting cytokine-based and chemokine inflammatory responses. This review covers implant debris-induced release of the cytokines and chemokines due to activation of the innate (and the adaptive) immune system and how this leads to subsequent implant failure through loosening and osteolysis, i.e., what is known of central chemokines (e.g., IL-8, monocyte chemotactic protein-1, MIP-1, CCL9, CCL10, CCL17, and CCL22) associated with implant debris reactivity as related to the innate immune system activation/cytokine expression, e.g., danger signaling (e.g., IL-1β, IL-18, IL-33, etc.), toll-like receptor activation (e.g., IL-6, tumor necrosis factor α, etc.), bone catabolism (e.g., TRAP5b), and hypoxia responses (HIF-1α). More study is needed, however, to fully understand these interactions to effectively counter cytokine- and chemokine-based orthopedic implant-related inflammation.

## Introduction

Total hip and knee replacements are examples of incredibly successful medical technologies with overall success rates of >90% at 10 years after surgery (1). However, the rate of failure grows with increasing time after surgery, where survival rates at 15–20 years post-op are very low at less than 50%. Currently, greater than 40,000 hip arthroplasties are revised each year in the US because of non-infection (aseptic)-related implant failure (painful implant loosening), and this is expected to increase by approximately 140% for total hip and 600% for total knee revisions over the next 25 years (1). Painful loosening is a serious long-term complication because of the risks of clinical/surgical of revision surgery.

Implant debris-induced biological reactions have been well established as the central cause of long-term implant failure (2, 3). However, other mechanisms of long-term implant failure have also been shown to contribute to the pathogenesis of implant failure, such as high fluid pressures forcing fluid between the bone and implant, endotoxin contamination (lipopolysaccharide from Gram-negative bacterial membranes), stress shielding where reduced stresses imposed on bone leads to decreased remodeling (4). Various mechanical factors, such as micromotion, may play a role in the induction of aseptic loosening not only directly but also indirectly through the formation of additional implant debris such as wear particles. Aseptic implant failure due to inflammation is responsible for >70% of total hip arthroplasty revisions and >44% of total knee arthroplasty revisions (2, 5). Local bone loss (or peri-implant osteolysis) is initiated by inflammatory responses to innate immune system interactions with small implant wear particles (generally <10 µm in diameter) resulting in persistent cytokine- and chemokine-induced inflammation in the peri-implant milieu (6). The focus of this review will be the identification of the central chemokines and cytokines involved in these innate and adaptive inflammatory reactions to implant debris (e.g., wear particles and metal ions).

## Innate Immune System Response to Wear Debris Particles

### Macrophages

**Figure 1:**
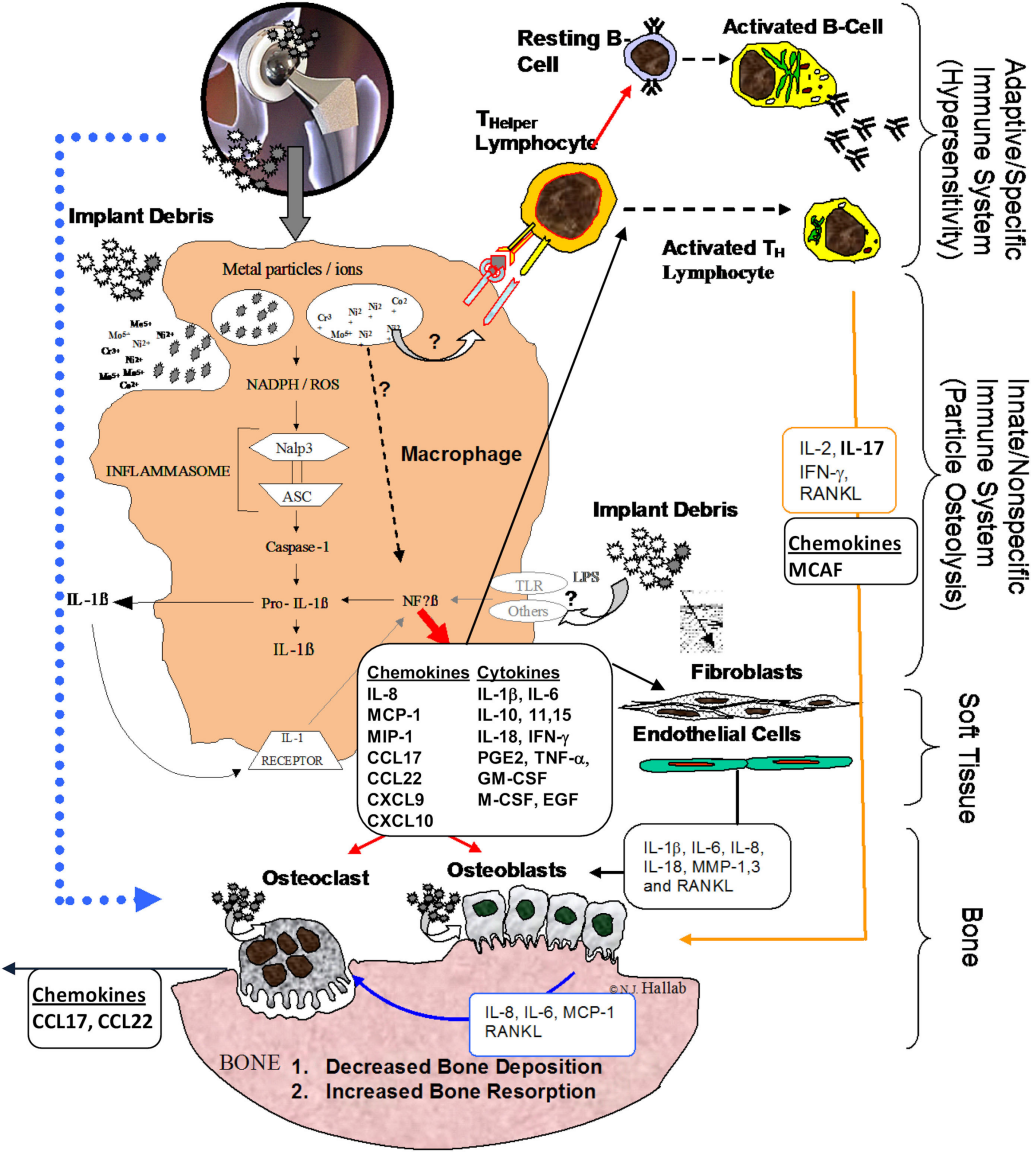
**Schematic of how the innate immune responses particularly inflammasome danger signaling is central to chemokine and cytokine implant debris-induced local inflammation and the pathology of implant loosening/failure (courtesy of Bioengineering Solutions Inc.)**.

Innate immune implant debris-induced inflammation is caused predominantly by macrophages, which react to aseptic (non-infected) implant debris upregulating pro-inflammatory transcription factors (e.g., NF-κB) and secreting inflammatory chemokines such as IL-8, monocyte chemotactic protein-1 (MCP-1), and MIP-1, and cytokines such as IL-1β, tumor necrosis factor α (TNF-α), and IL-6 (7) (Figure 1). Anti-inflammatory cytokines such as IL-10 modulate this inflammatory process, but how and which anti-inflammatory cytokines and chemokines dominate remains largely unknown.

Non-pathogenic-derived stimuli have been found to activate immune cells *via* danger signal pathways (8–11). This “inflammasome” pathway senses and transduces “danger-associated molecular patterns” (12) such as implant debris into an inflammatory response (13, 14). Other non-biological-derived danger signals include such cell damaging stimuli as UV light and particulate adjuvants present in modern vaccines (15, 16).

When particles activate the inflammasome pathway, cells release mature IL-1β, IL-18, IL-33, and other cytokines and chemokines as follows:

$$\begin{array}{l} \text{Debris}\to \text{Phagocytosis}\to \text{Lysosome damage}\to \text{ROS (reactive}\\ \text{oxygen species)}\to \text{Inflammasome (NALP3/ASC)}\to \text{Caspase1}\to \text{IL-}\\ \text{1β (and other IL-1-family) cytokines and chemokines (MCP-1, etc.).} \end{array}$$

Once phagocytosed by APCs such as macrophages, particles, such as asbestos and implant debris, induce danger signaling through mechanisms such as lysosomal destabilization. This lysosomal destabilization then causes a cascade of NADPH (nicotinamide adenine dinucleotide phosphate-oxidase), and an associated increase in reactive oxygen species, which then activates the intracellular multi-protein “inflammasome” complex composed of NALP3 (NACHT-, LRR-, and pyrin domain-containing protein 3) in association with ASC (apoptosis-associated speck-like protein containing a CARD domain) (17, 18). This inflammasome activation then activates Caspase-1, which does not act as an apoptosis stimulus (despite its caspase nomenclature) but rather converts cytokines such as IL-1β and IL-18 (and others) from their inactive into their active form. Recent studies demonstrate a polarization toward an M1 phenotype for macrophages in response to implant debris challenge (released metal ions and particles) (Figure 1) (19). Thus, given that wear particles are biologically active and influence the innate immune pathway, the amount, appearance, rate of production, time of exposure, and antigenicity of the wear particulates (and their breakdown products) are all important factors (8, 20). The macrophage M1-associated cytokines released after contact with wear debris include IL-1α, IL-1β, IL-6, IL-10, IL-11, IL-15, TNF-α, transforming growth factor α, granulocyte-macrophage colony-stimulating factor (GM-CSF), macrophage colony-stimulating factor (M-CSF), platelet-derived growth factor, and epidermal growth factor (Figure 1) (21–23).

## Adaptive Immune Responses

### Lymphocytes

All metal implants release implant debris through wear and corrosion (24, 25) and the released metal ions, while not sensitizers on their own, can act as haptens, activating the immune system by forming complexes with native proteins (26–28). Nickel is the most common delayed type hypersensitivity (DTH) sensitizer in humans followed by cobalt and chromium (29–32).

**Figure 2:**
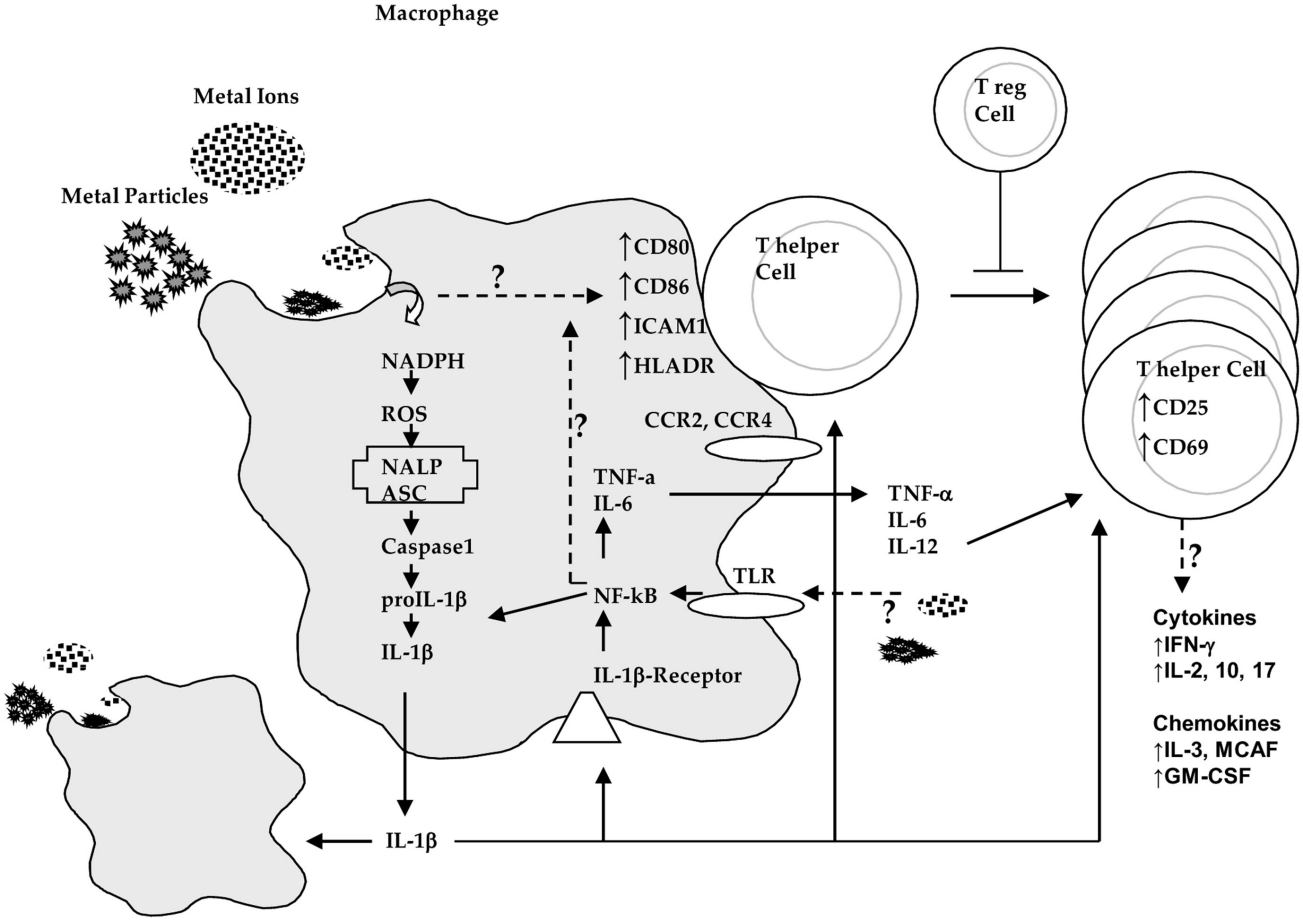
**Innate immune system (i.e., macrophage) interactions with implant debris produces danger signaling (inflammasome) and pathogen (NF-κB)-associated cytokines such as IL-1β and tumor necrosis factor α (TNF-α) and increased expression of costimulatory molecules such as CD80/86, ICAM1, and HLADR where the effects on chemokine receptors such as CCR2 and CCR4 are incompletely understood**. These innate responses can trigger adaptive immune responses where destructive TH1 type cytokine profiles that then require T-regulatory cells (e.g., IL-10) to control this response (courtesy of BioEngineering Solutions Inc.).

Lymphocytes have been shown that they can play a central role in the failure of some kinds of orthopedic implants (33–36). The subtypes of T-cells that dominate implant debris-associated responses are T-helper (TH) cells (33–36). These TH responses have been characterized as a type IV DTH response. DTH response to metal implant debris is an adaptive slow cell-mediated type of response. Metal-antigen sensitized and activated DTH T-cells release various chemokines, which recruit and activate macrophages [Figure 2; (37)] such as IL-3 and GM-CSF (promotes hematopoesis of granulocytes); monocyte chemotactic activating factor (promotes chemotaxis of monocytes toward areas of DTH activation); IFN-γ and TNF-β (produce a number of effects on local endothelial cells facilitating infiltration); and migration inhibitory factor (signals macrophages to remain in the local area of the DTH reaction). A DTH self-perpetuating response can create extensive tissue damage. Forms of metal sensitivity testing such as lymphocyte transformation test and patch testing (for skin reactions) are the only means to predict/diagnose those individuals that will have an excessive immune response to metal exposure that may lead to premature implant failure (approximately >1–2% patients/year) (37).

TH1 cells have been implicated as mediating metal DTH responses as characterized by production of IFN-γ and IL-2 and to a lesser degree IL-17. DTH response-associated chemokines fractalkine and CD40 indicate the possibility of TH17 activity (vs non-observed TH2 cell-mediated IL-10 responses) (36, 38). However, the chemokines involved in TH1 responses such as MIG (monokine induced by gamma interferon, i.e., CXCL9) and CXCL10 (39) have not been investigated in the context of adaptive immune responses to implant debris and greater understanding of their roles is critically needed. Specific lymphocyte responses (e.g., TH1 cells) may be underestimated and falsely attributed to innate immune responses because relatively very few activated lymphocytes locally can release macrophage-associated chemokines. It has been difficult to readily identify these responses in peri-implant tissues, by such signature cytokines as IL-2, interferon-γ, TNF-α, and IL-2 receptors (40). But some studies using mRNA detection instead of tissue immunohistochemistry (IL-2) have shown the increased expression of these TH1 cytokines (38).

### Bone Responses

#### Osteoclasts

The role of osteoclasts has been purported to be central to osteolysis, as they are the primary bone-resorbing cells. RANK(L) signaling is central for the activation of osteoclasts and activates a variety of downstream signaling pathways required for osteoclast development, but cross talk with other signaling pathways also fine-tunes bone homeostasis both in normal physiology and disease (41, 42). The degree to which other cells with the potential to resorb bone (e.g., macrophages) dominate implant debris-induced osteolysis remains controversial. The roles of released cytokines such as TNF-α are important to bone-related diseases (43), but their relative contribution to bone loss due to potent macrophage activation vs that of osteoclast activation alone, in implant debris-induced osteolysis, is not completely understood.

Osteoclasts (*in vitro*) have been shown capable of phagocytosing a wide size range of ceramic, polymeric, and metallic wear particles. After particle phagocytosis, they remain fully functional, hormone responsive, bone-resorbing cells (44, 45). However, we have reported that when fully differentiated *in vitro*, osteoclasts lose the ability to release inflammatory cytokines (46), thus indicating a diminished role for osteoclasts in recruiting and potentiating implant debris-induced inflammation and perhaps osteolysis as well.

#### Osteoblasts

Osteoblasts have shown the potential when stimulated *in vitro* by wear particles to produce osteoclastogenesis factors RANKL and M-CSF and cytokines such as IL-6 and IL-8 as well as VEGF. These *in vitro* investigations also demonstrated debris-induced decreased *de novo* synthesis of type 1 collagen as well as increased expression of matrix metalloproteinase 1 (MMP-1) (47–50). The caveat here is the important limitation “*in vitro* studies” and thus the degree to which osteoblasts are able to transduce implant debris stimuli into an inflammatory or functional effects is less well established *in vivo*.

### Soft Tissue Responses

#### Fibroblasts

Soft tissue cells such as fibroblasts are also actively involved in osteoclastogenesis and bone resorption (51, 52). The most prominent fibroblasts responses to implant wear debris were MMP-1, MCP-1, IL-1β, IL-6, IL-8, cyclooxygenase 1 (cox-1), cox-2, leukemia inhibitory factor, transforming growth factor beta 1, and TGFβ receptor type I. Additionally, downregulation of bone maintenance regulator such as osteoprotegrin (OPG) has been reported to decrease in osteoblasts/soft tissue cells exposed to implant debris and may contribute to regulatory RANKL/OPG imbalance in bone homeostasis contributing to the pathogenesis of implant debris-associated aseptic loosening/bone loss (53).

### Toxicity Responses

Toxicity responses are another facet of innate immune activation where apoptosis and hypoxia responses have been found to be induced by implant debris (54). While there is a plethora of reports by us (55–57) and others (58) implicating implant metals as “toxic” at high (and possibly clinically relevant) concentrations, there is little in terms of mechanism specificity, i.e., how implant metals induce this toxicity or what type of toxicity responses happen first. Additionally, confusing is the misidentification of metal ion-induced apoptosis rather than the more accurate pyroptosis (inflammatory apoptosis) when inflammatory cytokines have been identified. One specific mechanism that has been identified has been that of metal-induced hypoxia-like responses (54). Soluble and particulate metal debris have been shown to induce hypoxia-like pathology resulting in HIF-1α compensatory responses to metal implant debris by promoting both the induction of hypoxia (HIF-1α) and tissue angiogenesis (VEGF) providing a specific mechanism, which explains why local soft tissue growths (fibro-pseudotumors) and apoptosis responses can form in some people with certain orthopedic implants (54). The induction of apoptosis-like responses associated with implant debris has also been correlated with implant debris *in vivo*, such as caspase-3 associated with macrophages, giant cells, and T-lymphocytes in local tissues (capsules and interfacial membranes) of patients with aseptic hip implants (59). But, it is important not to confuse apoptosis with that of danger signaling and other inflammatory pathways because early studies using pan-caspase inhibitors (which inhibit danger signaling) erroneously concluded that inhibition of apoptosis by a pan-caspase inhibitors mitigates implant-induced inflammation osteolysis (60), when in fact it was the pan-caspase inhibition of inflammation pathways that decreased inflammation (8, 11). The role of apoptosis, pyroptosis, and pyronecrosis in implant-induced inflammation is still unclear and controversial.

## Central Chemokines in Implant Debris-Induced Inflammation

Chemokine expression by macrophages, fibroblasts, and osteoblasts exposed to implant debris is also a central innate immune effector reaction to implant debris enhancing migration to and inhibiting migration away from the site of implant debris (23, 61). The roles of chemokines relevant to the context of orthopedic implant debris include pro-inflammatory cytokine production, pyroptosis, apoptosis, angiogenesis, and collagen production, which act together to product aseptic bone resorption around implants. However, mostly macrophages and MSCs have been implicated as the major source of this chemokine in periprosthetic tissues induced by different types of wear particles like titanium, CoCr, and UMHWPE (62, 63). This migration of macrophages and osteoclasts to the sites around implants leads to accelerated osteolysis (64). The chemokines, particular to implant aseptic loosening pathology, include IL-8, MCP-1 MIP-1α, CCL17/thymus and activation-regulated chemokine (TARC), and CCL22/monocyte-derived chemokine (MDC) (64), which have been identified in peri-implant tissues and associated with implant debris reactivity (65–67).

### IL-8

IL-8, a CXC chemokine, is released by peri-implant cells such as macrophages, epithelial cells, MSCs, mast cells, and endothelial cells. It has been well established as present in periprosthetic tissues with implant debris and has been put forward as a biomarker of peri-implant osteolysis (47, 68, 69). Surprisingly, implant debris can induce the production of IL-8 by human osteoblasts (47, 70, 71). However, the main effector cells producing IL-8 are human macrophages that have migrated to the site of implant debris-induced inflammation (63). IL-8 attracts activated macrophages and neutrophils (PMNs) and which together with osteoclasts act to over ride the balance of bone homeostasis resulting in bone loss over time. However, the degree to which IL-8-dependent neutrophil attraction and activation affects implant–bone integrity over time is not clear. This may be due to the difficulty in modeling this system *in vitro*.

### Monocyte Chemotactic Protein-1

Increased expression of chemokines MCP-1 (CCL2), MIP1a (CCL3), and MIP 1α (CCL4) was observed in local tissues around failed arthroplasties and also produced by macrophages in cell culture after exposure to different types of wear particles (72). In contrast to MIP1α, an increased release of MCP-1 was also observed from fibroblasts after exposure to titanium and PMMA particles (73). MCP-1 (CCL2) potently chemoattracts monocytes but can also recruit macrophages, natural killer cells (NK cells), and T cells through the CCR2 or CCR4 receptors (74, 75). MCP-1 is produced by fibroblasts, osteoblasts, monocytes, and macrophages (74, 75). Thus as expected, implant debris can induce the production of MCP-1 in human fibroblasts, osteoblasts, monocytes, and macrophages together recruiting innate immune reactivity [i.e., monocytes and macrophages; (72, 73)]. MCP-1 has been found in peri-implant tissues of failed total joint implants, highlighting the potential of MCP-1 as potential biomarker of inflammation and osteolysis (72, 76). Implant debris particles such as PMMA or UHMWPE particles increased MCP-1 expression in RAW 264.7 macrophage cells (77, 78) where supernatant from particle-challenged macrophages caused THP-1 macrophages to migrate and was neutralized with the addition of antibody to MCP-1 (77, 78). While there has been some controversy as to whether blocking MCP-1/CCR2 interaction is effective at blocking macrophage recruitment *in vitro* (78), *in vivo* studies have shown that injected MCP-1 in a murine femoral implant model resulted in exogenous macrophage recruitment (RAW 264.7 cells) to the site of injection when challenge with of UHMWPE particles and that inhibiting the interaction of MCP-1/CCR2 decreased macrophage migration (22). However, while the use of injected CCR2-deficient macrophages resulted in less recruitment to the site of particle and MCP-1 challenge, there was still recruitment, demonstrating the pleiotropic nature of other CCRs and chemokines (79). However, the role of MCP-1 may be more complex. Kim et al. reported blocking MCP-1-induced formation of TRAP(+)/CTR(+) multinuclear cells was critical to blocking bone resorption (80). These findings show that MCP-1 is a potent chemokine involved in the complex pathology of osteolysis. However, there is a lack of *in vivo* (human or animal) data to indicate that interruption of a single, albeit potent, chemokine receptor interaction (MCP-1/CCR2) will reverse or prevent particle-induced inflammation (that is danger signal based) and prevent any resulting osteolysis (without significant negative consequences) given the multitude of other powerful inflammatory cytokines involved in this process and detailed in the following sections.

### MIP-1

Other chemokines such as MIP-1 have a less clear role in implant debris-induced inflammation. MIP-1 (MIP-1α CCL3 and MIP1β CCL4) is produced by a variety of peri-implant cell types including adaptive (lymphocytes) and innate (monocytes and macrophages), and tissue (fibroblasts and epithelial) cells (81). MIP-1α is likely central feature of adaptive immune responses (T-cells and B-cells) to implant debris; but to date, little evidence has shown that MIP-1 is central to adaptive (DTH) type immune responses observed in peri-implant tissues with elevated metal debris (33, 35, 82). However, monocytes, neutrophils, dendritic cells, and NK cells are also effected by MIP-1, to foster adaptive immune responses (83, 84). *In vitro*, metal (titanium) and polymeric (PMMA) implant wear debris was found to increase the production of MIP-1α by primary human monocytes/macrophages, resulting in increased monocyte migration. Countering MIP-1 with a MIP-1 antibody decreased this migratory effect (72). However, these findings have been challenged by others where RAW 264.7 cells failed to produce increased amounts of MIP-1α when challenged with wear particles. Moreover, a neutralizing antibody to MIP-1α failed to inhibit the migration of THP-1 macrophages in culture challenged with implant debris particles (78). A lack of response was also found for MSCs during MIP-1/wear debris induction. Huang et al. found that using a neutralizing antibody to CCR1 (one of the receptors for MIP-1α) failed to affect the migration of MSCs challenged with implant debris particles *in vitro*. However, the actions of CCR1 involve many ligands (e.g., MIP-1α, MCP-3, and RANTES), and others have found that neutralizing the actions of CCR1 in the presence of particles challenge does indeed lead to a decrease of MSC migration and differentiation into osteoblasts (22). Thus, currently, there is insufficient evidence to indicating a central role for MIP-1α in pathology of implant debris-induced inflammation and osteolysis.

### CCL17 and CCL22

CCL17/TARC, CCL20/MIP-3alpha, and CCL22/MDC both interact with the chemokine receptor CCR4 and are important chemokines for adaptive immune responses (85). They are known to be mainly produced by cell lineages closely related to osteoclasts such as dendritic cells and are examples of chemokines that are produced in secondary lymphoid organs and in peripheral tissues (86). CCL22 and CCL17 are produced by macrophages, dendritic cells, and endothelial cells and act as adaptive immune chemokines affecting TH2 population, and are associated with allergy and dermal hypersensitivity to haptens when produced by keratinocytes and langerhans cells (39). These CCL17 and CCL22 chemokines have also been shown induced by the exposure of metal implant debris (e.g., titanium particles) on bone cells (osteoclasts and osteoblasts) (87). In addition, the receptors for these chemokines CCR4 were shown increased in macrophage-like osteoclasts precursor cells (87). Moreover, the expression of CCR4 was upregulated when osteoclast precursors were stimulated with titanium particles (87).

**Figure 3:**
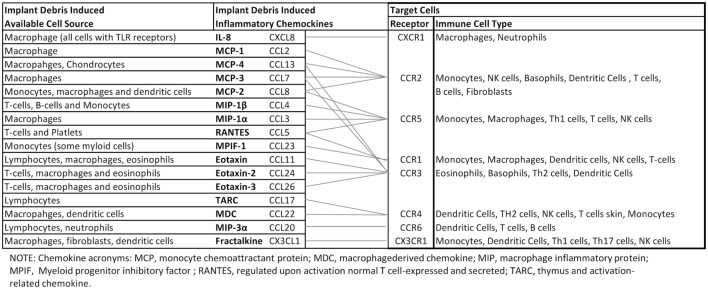
**Orthopedic implant debris act on a number of different cells around implants inducing the release of chemokines**. Different types of immune cells are recruited by different chemokines. However, there is crossover between the receptors associated with different ligand/chemokines. This schematic highlights the complexity associated with understanding, which key chemokines are best targeted for mitigating implant debris-induced inflammation (88–95).

Central chemokines to implant debris-induced inflammation and bone loss and their effects are summarized in Figure 3. Given the complexity of multiple receptors and chemokines involved, further study is required to understand the central mediators involved in the in the migration of MSCs sites of peri-implant inflammation.

## Conclusion

Implant debris-induced chemokine expression and the interplay between resulting chemokine and cytokine expression are incompletely characterized and currently limited to a basic understanding that a few central chemokines including MCP-1, IL-8, and MIP-1 are important. Central among these seems to be MCP-1. However, despite this centrality, it seems unlikely that interruption of only one pathway (e.g., MCP-1/CCR2) will be effective at mitigating implant debris-induced inflammation given the numerous responses detailed in this review and the pleiotropic nature of chemokines and chemokine receptors (e.g., MCP-1 binds to both CCR2 and CCR4, Figure 3). Additionally, it is important to note that chemokine response is essentially a downstream effect of debris-induced inflammation (i.e., cytokine induced) and thus the single bullet strategy of inhibiting a single chemokine to address aseptic inflammatory osteolysis is unlikely to succeed clinically as a useful strategy until more sophisticated understanding of this interplay is understood. It is important to note that most of our current understanding of cytokines, chemokines, and bioreactivity associated with implant debris-induced inflammation and aseptic loosening comes from *in vitro* models that may be overly simplistic. Continuing consensus-building *in vivo* investigations/evidence will be required to support current models/understanding.

The serious pathology of aseptic inflammation and resultant osteolysis around joint replacement implants is intimately dependent on both cytokines and chemokines released by innate and adaptive immune reactions and local cells around implants. These types of debris-induced inflammation are dominated by innate immune cell (macrophages) secretion of TNF-α, IL-1β, IL-6, and PGE2, which together with potent chemokines such as MCP-1 causes a persistent low-grade immune reaction resulting in peri-implant bone resorption. Given the increasing number of people receiving orthopedic implants, the issue of biologic reactivity is growing more critical. There is increasing need for more detailed study of implant debris-induced cytokine and chemokine interplay to mitigate this response effectively.

## Author Contributions

Both authors have participated in the writing of this review article.

## Conflict of Interest Statement

The authors declare that the research was conducted in the absence of any commercial or financial relationships that could be construed as a potential conflict of interest.
